# Repeated triplets complicated by monochorionic diamniotic twins following assisted reproduction: a case report and literature review

**DOI:** 10.1186/s12884-020-03055-y

**Published:** 2020-06-23

**Authors:** Bing Song, Zhe Wang, Yujie Chen, Chao Wang, Zhaolian Wei, Xiaojin He, Yunxia Cao

**Affiliations:** 1 Reproductive Medicine Center, Department of Obstetrics and Gynecology, the First Affiliated Hospital of Anhui Medical University, Hefei, 230032 China; 2 NHC Key Laboratory of Study on Abnormal Gametes and Reproductive Tract, Anhui Medical University, Hefei, 230032 China; 3 Ministry of Education Key Laboratory of Population Health Across Life Cycle, Anhui Medical University, Hefei, 230032 China; 4 Anhui Province Key Laboratory of Reproductive Health and Genetics, Hefei, 230032 China; 5 Biopreservation and Artificial Organs, Anhui Provincial Engineering Research Center, Anhui Medical University, Hefei, 230032 China

**Keywords:** Triplets, Monochorionic diamniotic twins, Assisted reproduction, Case report

## Abstract

**Background:**

Monochorionic twinning involves numerous maternal and fetal complications, triplets complicated by a monochorionic pair are at further increased risk. Here, we report a case of repeated triplets complicated by monochorionic diamniotic twins with successful pregnancy outcomes in a woman using autologous oocytes.

**Case presentation:**

A 30-year-old female undergoing embryo transfer with fresh and frozen embryo cycles with autologous oocytes. The two cycles were confirmed by transvaginal ultrasound to result in successful clinical pregnancies of triplets complicated by a monochorionic twinning. The first pregnancy resulted in a singleton delivery after a selective reduction of the monochorionic pair. The subsequent pregnancy resulted in a dichorionic diamniotic twin pregnancy after the heartbeat of one of the monochorionic twin fetuses stopped at 43 days after embryo transfer. Both of the pregnancies ended with successful live births.

**Conclusions:**

Our case report of repeated triplets with monochorionic twins suggests the potential causes and risk factors of monochorionic twinning in assisted reproduction and raises concern regarding the timing of multifetal pregnancy reduction.

## Background

Many studies have demonstrated that an increased risk of monozygotic twins(MZT) was associated with assisted reproductive techniques(ART) procedures [1–4]. According to published studies, the incidence of MZT after ART varies from 1.57 to 13.2% compared with the natural conception rate of 0.4% [1–5]. There are three types of monozygotic: dichorionic-diamniotic, monochorionic-diamniotic and monochorionic-monoamniotic. Monochorionic twin occupies most of the proportion of the incidence in monozygotic twinning. It is well-known that monochorionic twins are at high risk of maternal and fetal complications, including low birthweight, preterm birth, twin-twin transfusion syndrome(TTTS), umbilical cord accidents and perinatal mortality [6–9]. Regarding dichorionic triplets pregnancy, the rate was only 0.004% among all the pregnancies, as previously reported [10]. Here, we describe a rare case of repeated triplet pregnancy complicated by a monochorionic diamniotic twin in a patient after assisted reproduction. To the best of our knowledge, this is the first published report of this kind. This case highlights the risk factors and obstetric outcomes of dichorionic triplets.

## Case presentation

A couple who complained of secondary infertility for 6 years underwent IVF treatment due to polycystic ovarian syndrome and male ejaculatory dysfunction. The patient was a 30-year-old female, her cycle was followed with a standard long protocol for 18 days that included uFSH (150 units every day, Zhuhai, China) and HMG (75 units every day after 8th day of ovarian stimulation; Zhuhai, China). Final oocyte maturation was triggered by the administration of 10,000 IU of HCG (HCG 10000 U for injection; Zhuhai, China). Oocyte retrieval was carried out by the transvaginal ultrasound-guided puncture of follicles. Sixteen mature oocytes were retrieved under transvaginal ultrasound guidance, of which 13 were fertilized after intracytoplasmic sperm injection(ICSI). Finally, 7 good-quality embryos were retrieved. Embryo culture in vitro showed two 8-cell embryos (grade A) and two 9-cell embryos (grade B) on day 3. Other embryos were culture to day 5 and yielded 3 viable blastocysts.

**Fig. 1:**
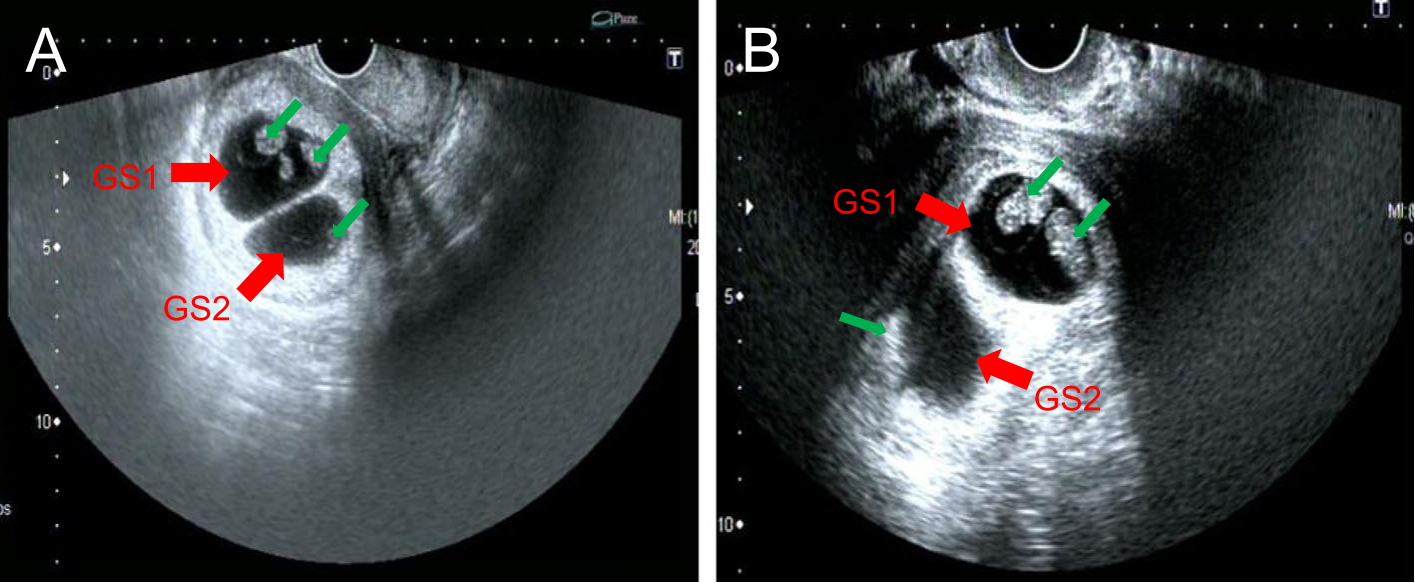
Dichorionic triplets were diagnosed by transvaginal ultrasound, two gestational sacs (GSs, red arrowhead) and three germs (green arrowhead) were detected inside the uterus. A) Ultrasound image of first pregnancy at 5 weeks post-transfer. B) Ultrasound image of second pregnancy on day 37 post-transfer

Two cleavage embryos (one 8-cell embryo and one 9-cell embryo) were transferred on day 3 in the fresh cycle. The other two day 3 embryos were cryopreserved. Luteal progesterone support was given after the oocyte operation. The patient had confirmed pregnancy by a urine pregnancy test on day 14 post-transfer. Dichorionic triplets were diagnosed by a transvaginal ultrasound scan at 5 weeks post-transfer, as two gestational sacs (GSs) inside the uterus and two pulsating echoes were detected in one of the GS (Fig. 1A). After counselling the maternal and fetal risks, the patient decided to have a single pregnancy with the help of multifetal pregnancy reduction(MFPR) for both monochorionic twins. The selective MFPR was successfully performed at 6 weeks post-transfer. The subsequent pregnancy went well without any complications. The patient had a full-term vaginal delivery at 41 weeks and 6 days. The birth weight and Apgar scores at 1 min and 5 min were 3800 g and 10–10.respectively.

When China’s two-child policy was loosened, the patient requested a frozen embryo transfer when she was 33 years old. Hormone replacement therapy (HRT) was used in the first frozen cycle. A single blastocyst (Gardner’s classification: 4AA) was transferred, and the transfer failed. During the next cycle, two day 6 embryos (Gardner’s classification: 4CB*2) was transferred, but the transfer failed again. The woman successfully conceived in the following cycle after two cleavage embryos (8 cells grade 2 and 9 cells grade 2) were transferred. We first suspected dizygotic twins, as two gestational sacs (GSs) was detected with transvaginal US on day 30 post-transfer. However, the US showed two GSs with three fetuses one week later, and another dichorionic triamniotic triplet pregnancy was suspected (Fig. 1B). The heartbeat of one of the monozygotic twins stopped suddenly on day 43 post-transfer, resulting in a dichorionic diamniotic twin pregnancy. Two healthy boys were delivered by emergency caesarean section at 35 weeks and 2 days of gestation due to premature rupture of membrane (PROM). Their birth weight and Apgar scores at 1 min and 5 min were 1900 g, 9–10 and 2800 g, 10–10, respectively. The lighter baby was sent to the neonatal care unit and was discharged 3 weeks after birth.

## Discussion and conclusions

The data showed that there may be a significantly elevated risk of monozygotic twins after ART compared to natural conception [2, 3]. Monochorionic twin occupies most of the proportion of the incidence in monozygotic twinning. According to our previous large retrospective cohort study, the overall incidence of monochorionic diamniotic twinning was 2.55% after ART [4]. It was difficult to identify the exact mechanisms responsible for embryonic splitting after ART [11]. Potential risk factors that lead to an increased incidence may be micromanipulation of the zona pellucida(ZP), blastocyst transfer, age of the oocyte, and embryo cohort quality. The cause has been investigated but is still controversial [12–23].

It is plausible that an artificial breach in the ZP caused by the ICSI, assisted hatching(AH) or preimplantation genetic diagnosis may cause splitting of the inner cell mass(ICM) and MZ twinning [15, 16]. However, recent studies have questioned the role of ZP manipulation, because these studies did not find an increased rate of MZT after the ICSI and AH [17]. Many studies have examined maternal age (or oocyte age) as a risk factor. Song et al. and Knopman et al. both observed a significant increase in the rate of MZT with younger maternal age and proposed that the age of the oocyte could be an important contributor to the incidence of MZT [4, 12]. This conclusion was confirmed in a systematic meta-analysis [13].

Recent studies reported that the transfer of a blastocyst resulted in a higher incidence of MZ pregnancies than the transfer of cleavage-stage embryos [18–22]. Our 6-year single center retrospective study demonstrated that younger maternal age and prolonged embryo culture might be potential contributing factors associated with a higher incidence of monochorionic diamniotic twinning in ART [4]. However, some researchers attributed this association to the high-quality embryos generated by prolonged embryo culture. In their opinion, blastocyst transfer is not associated with the incidence of MZT when controlling for embryo cohort quality [23].

It has been shown that multiple pregnancies are associated with an increased incidence of abortion and premature labor. Triplets complicated by a monochorionic twin are at further increased risk of many maternal and fetal complications [24]. A selective reduction of the monochorionic pair is usually recommended when using MFPR in the obstetric management of complex triplets [25, 26]. In the first pregnancy of this case, we had a good obstetric outcome (full-term normal delivery at 41 weeks + 6 days in this case). The transvaginal fetal reduction technique proved to be safe and effective, and it can be performed as early as 7 weeks of gestation. However, it should be noted that spontaneous reduction can occur before 12 weeks, and some scholars propose that selective reduction should be performed between 10 and 13 weeks when the nuchal translucency((NT) thickness and fetal abnormalities are easier to measure and diagnose [26, 27]. In the second successful pregnancy of the patient, the heartbeat in one of the monozygotic twin fetuses stopped at 43 days after embryo transfer, and the patient had a dichronic twin pregnancy. Guidelines or an expert consensus should be issued regarding the timing and decision to retain the singleton or twins when using MFPR in triplet pregnancies with monochorionic twins.

To our knowledge, this is the first reported case of repeated triplets complicated by a monochorionic pair in a patient with autologous oocytes following assisted reproduction treatment. Potential risk factors for this patient include young maternal age, high quality embryos and micromanipulation of the ZP. In the cases with dichorionic triplets, selective multifetal pregnancy reduction is associated with a better obstetric outcome and should be offered in the obstetric management. While these cases are no longer rare after an ART procedure, the potential causes and risk factors of monochorionic twinning in assisted reproduction and the timing of MFPR warrant continued investigation.

## Data Availability

The data referred to this case during the current study was available on reasonable request.

## Abbreviations

MZT: Monozygotic twins
ART: Assisted reproductive techniques
ICSI: Intracytoplasmic sperm injection
MFPR: Multifetal pregnancy reduction
ZP: Zona pellucida
HRT: Hormone replacement therapy
GSs: Gestational sacs
AH: Assisted hatching
